# CRISPR screening in cardiovascular research

**DOI:** 10.3389/fcell.2023.1175849

**Published:** 2023-04-12

**Authors:** Haihuan Shan, Teng Fei

**Affiliations:** ^1^ National Frontiers Science Center for Industrial Intelligence and Systems Optimization, Key Laboratory of Bioresource Research and Development of Liaoning Province, College of Life and Health Sciences, Northeastern University, Shenyang, China; ^2^ Key Laboratory of Data Analytics and Optimization for Smart Industry (Northeastern University), Ministry of Education, Shenyang, China

**Keywords:** CRISPR, screening, screen, cardiovascular, disease

## Abstract

The recent advent and widespread application of CRISPR-based genome editing tools have revolutionized biomedical research and beyond. Taking advantage of high perturbation efficiency and scalability, CRISPR screening has been regarded as one of the most powerful technologies in functional genomics which allows investigation of different genetic subjects at a large scale in parallel. Significant progress has been made using various CRISPR screening tools especially in cancer research, however, fewer attempts and less success are reported in other contexts. In this mini-review, we discuss how CRISPR screening has been implemented in studies on cardiovascular research and related metabolic disorders, highlight the scientific progress utilizing CRISPR screening, and further envision how to fully unleash the power of this technique to expedite scientific discoveries in these fields.

## 1 Introduction

Cardiovascular diseases and related metabolic disorders have been major threats for human health in modern societies (Zhao et al., 2015; Zhao et al., 2019). To develop effective therapeutics in a rational and efficient way, significant efforts are devoted to decoding the genetic basis and molecular mechanisms behind these pathological processes. Traditional genetic perturbation tools such as gene targeting by homologous recombination, overexpression of complementary DNA (cDNA) and RNA interference (RNAi) are widely employed to investigate gene functions. Particularly, high-throughput genetic screening using RNAi technology in either individually array-based or pooled format has led to expedited discovery of functional genes in cardiovascular research such as cholesterol transport, vascular physiology and heart development (Bartz et al., 2009; Neely et al., 2010; Chu et al., 2015; Kraehling et al., 2016; Xu et al., 2018). However, the efficiency and applicability of RNAi are not always satisfactory especially for those hard-to-transfect primary cells and advanced animal models in cardiovascular research. Such limitation has been largely resolved as the introduction and application of clustered regularly interspaced short palindromic repeats (CRISPR)-based genome editing tools (Wang and Doudna, 2023). The simplicity, high efficiency, and scalability of CRISPR techniques have revolutionized many areas of biomedical research including cardiovascular field (Strong and Musunuru, 2017; Musunuru, 2022; Nishiga et al., 2022). This mini-review aims to summarize how CRISPR screening is applied in cardiovascular research and outlook its future directions.

## 2 CRISPR technology and high-throughput screening

**FIGURE 1:**
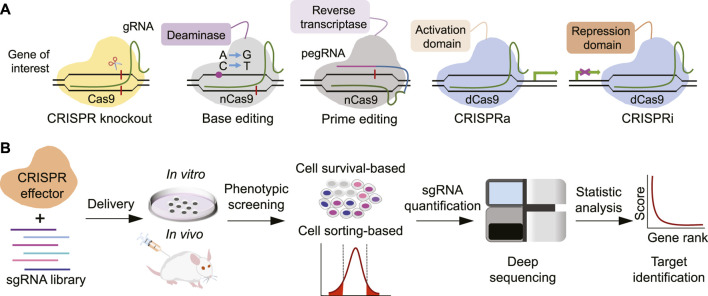
Overview of CRISPR technology. **(A)** Major modules of CRISPR toolbox. **(B)** Workflow of typical CRISPR screening experiments.

The natural CRISPR-Cas systems are present in most bacteria and provide acquired immunity against invaded viruses or pathogens. Leveraging its capability to destroy or interfere with genetic materials, scientists have adapted CRISPR-Cas systems into powerful genome editing tools (Wang & Doudna, 2023). The typical CRISPR-Cas9 system comprises two basic components: a DNA-cutting nuclease protein Cas9 and a single stretch of RNA molecule called single guide RNA (sgRNA). The sgRNA is a concatemer of a 17–20 nucleotide target DNA-matching CRISPR RNA (crRNA) and a trans-activating CRISPR RNA (tracrRNA) which provides binding scaffold for Cas nuclease. Once the sgRNA locates its targeted DNA sequence, the associated Cas9 nuclease produces a double-stranded DNA break (DSB) at specific position adjacent to protospacer adjacent motif (PAM). Non-homologous end-joining (NHEJ)-mediated DNA repair may generate indels at targeted loci which will likely disrupt normal protein coding if the indels are within the open reading frame. Such CRISPR-mediated gene knockout is highly efficient as a loss-of-function method for a protein-coding gene. When provided with a DNA repair template containing an intended editing sequence, precise genome editing can be achieved *via* the homology-directed repair (HDR) mechanism (Hsu et al., 2014). In addition to Cas9, other CRISPR effectors have been continuously discovered and repurposed as DNA or RNA editing tools such as Cas12a and Cas13 (Zetsche et al., 2015; Abudayyeh et al., 2017). Another important category of CRISPR-based genome editing tool is base editor which usually employs a Cas9 nickase (nCas9) fused to a nucleobase deamination enzyme and allows precise single nucleotide correction without inducing DSB and requirement of a repair template (Rees & Liu, 2018). Such precision editing is advantageous in correcting pathologic single-nucleotide variant (SNV) and holds great potential for treating human genetic disorders in clinics. Recently, another CRISPR-based precision editing tool called prime editor has also been developed which is composed of a nCas9 fusion with a reverse transcriptase (RT) and a prime editing gRNA (pegRNA) simultaneously specifying target site and template with the desired edit (Anzalone et al., 2019). Although prime editing enables virtually any base substitution and small insertion/deletion without introducing DSB, further optimization is essentially required to increase its editing efficiency and lower the unwanted edits. In addition to genome editing, the CRISPR-Cas systems are also adapted to control gene expression with tools such as CRISPR interference (CRISPRi) and CRISPR activation (CRISPRa) or to monitor specific molecules by imaging techniques (Dominguez et al., 2016) (Figure 1A).

The engagement of above CRISPR-based tools significantly boosts the capacity of genetic perturbations, especially when combining with high throughput screening platforms (Bock et al., 2022). The ease of synthesizing and cloning massive sgRNA oligos enables researchers to perform CRISPR-based genetic screens in either arrayed or pooled format on targeted cells or model systems. For a typical pooled CRISPR screen, designed sgRNA oligos are firstly synthesized in a chip and then collectively cloned into a lentiviral expressing vector to construct a plasmid library. These sgRNA-expressing cassettes are introduced into targeted cells *via* lentiviral infection. The samples are collected before and after the infliction of selection pressure associated with the process of interest by either direct collection of viable cells or flow cytometry-based sorting of specific cells. The genomic DNA is then extracted to amplify integrated sgRNA element followed by quantification with high throughput sequencing. The identity of targeted genetic subject producing significant perturbation effect can be retrieved by the statistic changes of corresponding sgRNA abundance (Figure 1B). Such CRISPR screens have been widely applied in diverse scenarios especially cancer research and exhibit magnificent power to pinpoint key genetic players or dissect genetic interactions for investigated processes (Bock et al., 2022). The implementation of CRISPR screens in cardiovascular research is just beginning and has already led to several important findings.

## 3 CRISPR screening in cardiovascular research

### 3.1 Cholesterol metabolism and atherosclerosis

Hypercholesterolemia is a major risk factor for atherosclerotic cardiovascular diseases and fine-tuning of cholesterol level in the blood represents one of the mainstream preventive and therapeutic interventions. Unraveling the molecular mechanisms behind cholesterol homeostasis has been the central question in this field. To systematically investigate the key gene regulators that control cellular uptake of circulating low-density lipoprotein (LDL) cholesterol, Emmer et al. performed a genome-wide CRISPR knockout screen in human liver-derived cell line Huh7 (Emmer et al., 2021). Fluorescently labeled LDL was used to represent LDL cholesterol and gene-edited cells with significantly high or low fluorescent LDL uptake were sorted by flow cytometry. In addition to known regulators such as *LDLR*, *SCAP* and *MBTPS1*, they identified tens of genes that can either enhance or decrease LDL uptake. By performing CRISPR screen in LDLR-depleted cells using a focused library, they further pinpointed several genes with LDLR-independent roles during LDL uptake. Furthermore, they also found tens of genes that regulate total or cell surface level of LDLR by sorting LDLR-stained cells in a focused CRISPR screen (Emmer et al., 2021). One of screening hit RAB10, a small GTPase, was later shown in a follow-up study to promote LDLR recycling from RAB11-positive endosomes to the plasma, thereby providing a mechanistic explanation on its role in LDL cholesterol uptake (Khan et al., 2022). Interestingly, the function of RAB10 on LDL cholesterol regulation was further confirmed by another recent study in which several CRISPR screens were conducted with different focused libraries in human liver HepG2 cells to identify genes affecting LDL cholesterol uptake. Similar readout and cell sorting strategy were employed, however, this study mainly focused on those genes adjacent to variants associated with serum LDL cholesterol from human Genome-Wide Association Study (GWAS) data, and CRISPRa screen was also employed as an orthogonal approach. In this way, they found that 21 genes with human genetic evidence may regulate LDL cholesterol uptake including *RAB10* and *OTX2* (Hamilton et al., 2023). Another group performed similar CRISPR screen in HepG2 cells at whole genome scale to explore genes whose loss-of-function compromises LDL cholesterol uptake, and determined transgelin (encoded by *TAGLN*) as a novel hit which acts by disrupting actin-dependent clathrin-mediated endocytosis of LDL (Lucero et al., 2022). Despite similar readout and targeted cells, these studies adopted different but orthogonal gene perturbation approaches (e.g., CRISPR knockout, CRISPRa and CRISPRi) with varied screening libraries. These efforts are complementary to each other and collectively provide a wealth of resources for understanding the molecular basis of cholesterol transport.

CRISPRi-based genome-scale screen was also implemented to study gene regulators of cell surface LDLR level in HepG2 cells. Cold shock domain-containing protein E1 (encoded by *CSDE1*) was shown to be an interesting hit with therapeutic potential against hypercholesterolemia by negatively regulating *LDLR* mRNA stability in a post-transcriptional manner (Smith et al., 2022). In addition to LDLR itself, genome-wide CRISPR knockout screen was also performed to identify genes that affect PCSK9, a well-known negative regulator of LDLR and therapeutic target to lower LDL cholesterol. Using intracellular accumulation of PCSK9 as a readout in cell sorting-based screening system, an endoplasmic reticulum (ER) cargo receptor called SURF4 was proved to be essential for efficient cellular secretion of PCSK9 in HEK293T cells (Emmer et al., 2018). HMG-CoA reductase (HMGCR), the rate-limiting enzyme of the cholesterol biosynthesis, also stands central in maintaining cholesterol homeostasis. CRISPR knockout screen in HeLa cells engineered to express a sterol-responsive HMGCR-fluorophore fusion protein led to identification of genes that regulate HMGCR protein stability. A novel E3 ligase RNF145 was shown to mediate HMGCR degradation in concert with gp78 and Hrd1 E3 ligase (Menzies et al., 2018). Furthermore, Xiao et al. carried out a cell survival-based CRISPR knockout screen to identify key regulators of cholesterol homeostasis and revealed a previously uncharacterized protein POST1(encoded by *C12ORF49*) as an important factor to control SREBP signaling and S1P maturation (Xiao et al., 2021).

Cholesterol transport between endosome, lysosome, ER, lipid droplet and plasma membrane within the cell must undergo delicate regulation for proper cholesterol and lipid homeostasis. Several groups have applied CRISPR screen to elucidate regulators during these processes. Using an engineered SREBP2-dependent cholesterol reporter line of HeLa cells, hundreds of genes were catalogued by a genome-wide CRISPR knockout screen for orchestrating cholesterol transport, and the trimeric Mon1-Ccz1-C18orf8 guanidine exchange factor for Rab7 was shown to control NPC1-dependent lysosomal cholesterol export (van den Boomen et al., 2020). Using a membrane-anchored LDLR level as readout, another genome-scale CRISPR knockout screen performed in human fibroblast SV589 cells identified PTDSS1, an enzyme that synthesizes a phospholipid component of plasma membrane called phosphatidylserine, as a critical factor for cholesterol trafficking between lysosome, ER and plasma membrane (Trinh et al., 2020). PTDSS1 was also independently discovered in another CRISPR knockout screen aiming to identify endolysosomal cholesterol regulators and this study further characterized the roles and mechanisms of ER-localized SNX13 as a negative regulator of lysosomal cholesterol export (Lu et al., 2022).

### 3.2 Screening in blood and vascular cells

Many researchers have applied CRISPR screening in immune cells to investigate questions in tumor immunology (reviewed elsewhere) (Li & Fei, 2020; Shi et al., 2022), but very few studies are linked to cardiovascular diseases. Inflammation plays a prominent role in the development of cardiovascular diseases such as atherosclerosis which involves inflammation-induced endothelial dysfunction and interplay between blood vessel cells and inflammation-related immune cells (e.g., monocytes and macrophages) (Henein et al., 2022). Using immortalized murine macrophages and Nlrp3-dependent cell death as a readout, Schmid-Burgk et al. described a genome-scale CRISPR screen, and pinpointed NEK7 as a critical player for NLRP3 inflammasome activation which underlies many metabolic diseases including atherosclerosis (Schmid-Burgk et al., 2016). Jimenez-Duran et al. performed a genome-wide CRISPR knockout screen in human monocytic cell line THP-1 to identify key genes that regulate CD14 expression and macrophage differentiation. Using CD14 expression level as cell sorting criteria, this screen uncovered several genes to be involved in the above processes and they validated an inhibitor of screening target MAP2K3 for effectively preventing inflammatory cytokine production from activated primary macrophages (Jimenez-Duran et al., 2020). Another study employed a murine immortalized myeloid precursor cell line ER-Hoxb8 for a genome-scale CRISPR knockout screen to explore the regulators for proinflammatory alarmins S100A8 and S100A9. They found a critical role of transcription factor C/EBPδ to control S100A8/A9 expression in murine monocytes (Jauch-Speer et al., 2022). Given the importance of inflammation in cardiovascular diseases, these efforts are of particular interests and demonstrate the power of CRISPR screen to study such complex diseases.

As key components of vascular system, blood endothelial cells (ECs) and their related processes have been closely associated with cardiovascular diseases. Using an immortalized microvascular blood EC line cultured in a 3D microcarrier-based system, He et al. carried out a kinome-wide CRISPR knockout screen to uncover kinase regulators that modulate EC response to anti-angiogenic therapy - bevacizumab, a neutralizing monoclonal antibody against vascular endothelial growth factor A (VEGFA). This cell viability-based screen revealed important functions of the bromodomain and extra-terminal domain (BET) family of proteins BRD2/3/4 in regulating EC survival and response to VEGFA blockade (He et al., 2021). Another study applied genome-wide CRISPR knockout screen in an immortalized mouse aortic endothelial cell line to investigate the molecular underpinnings of EC cells in response to laminar shear stress (LSS) from blood stream. Using LSS-induced key transcription factor KLF2 as screening readout, this study revealed a mitochondria-involved MEKK2/3-MEK5-ERK5 regulatory axis that underlies LSS induction of KLF2 (Coon et al., 2022).

### 3.3 Heart development and regeneration

A genome-wide CRISPR knockout screen was initially deployed to identify genetic regulators that determine the reprogramming efficiency of murine cardiac fibroblast into cardiac progenitors by a chemically induced approach. Loss of DNA methyltransferase 1-associated protein 1 (Damp1) was shown to enhance Nkx2-5-positive cardiac progenitor conversion and affect further differentiation of these progenitor cells (Yu et al., 2019). Another study created CRISPR-mediated mutant library for more than 6,000 genes in human embryonic stem cells and allow these cells to differentiate into MESP1-positive cardiac mesoderm or ISL1-positive cardiac progenitor cells. A total of 15 candidate genes including ZIC2 were identified to control these cell fate transitions (Xu et al., 2020). VanDusen et al. further designed an *in vivo* CRISPR knockout screening system in newborn mice to study cardiomyocyte maturation by adeno-associated virus (AAV)-mediated introduction of sgRNA library into the myocardium and flow cytometry-based marker gene selection. Among more than 2,000 tested genes, they discovered several transcriptional regulators such as RNF20 and RNF40 to be pivotal players during cardiomyocyte maturation (VanDusen et al., 2021). Pettinato et al. introduced a CRISPR knockout library into an engineered human induced pluripotent stem cell (iPSC) line with cyclin B1 (CCNB1) as a reporter to study human cardiomyocyte replication and polyploidization in iPSC-derived cardiomyocytes. They uncovered p53 as a driver of cardiomyocyte polyploidization by inhibition of CCNB1 (Pettinato et al., 2021). Genome-wide CRISPR knockout screen has also been performed in human iPSCs and their derived cardiomyocytes to determine gene mediators of drug cardiotoxicity. Using cell viability as screening readout, Sapp et al. identified human-specific transporters SLCO1A2 and SLCO1B3 whose loss-of-function protects against doxorubicin-induced cardiotoxicity (Sapp et al., 2021). Furthermore, Parvez et al. developed a droplet microfluidics-based platform to allow *in vivo* CRISPR screening in zebrafish at embryo or animal level. Using this system, they identified several hits out of 188 poorly characterized genes which are important for cardiac development and function (Parvez et al., 2021) (Table 1).

**TABLE 1:** List of pioneer studies performing CRISPR screen in cardiovascular research.

CRISPR module	Target cell	Screen mode	Screen aim	Reference
knockout	Huh7	*in vitro*	cellular uptake of circulating LDL cholesterol	Emmer et al. (2021)
knockout/activation	HepG2	*in vitro*	LDL cholesterol uptake	Hamilton et al. (2023)
knockout	HepG2	*in vitro*	LDL cholesterol uptake	Lucero et al. (2022)
interference	HepG2	*in vitro*	cell surface LDLR level	Smith et al. (2022)
knockout	HEK293T	*in vitro*	cellular secretion of PCSK9	Emmer et al. (2018)
knockout	HeLa	*in vitro*	post-translational regulation of HMGCR	Menzies et al. (2018)
knockout	HeLa	*in vitro*	cellular cholesterol homeostasis	Xiao et al. (2021)
knockout	HeLa	*in vitro*	cholesterol homeostasis and transport	van den Boomen et al. (2020)
knockout	SV589	*in vitro*	cholesterol trafficking	Trinh et al. (2020)
knockout	K562	*in vitro*	level of lysosomal cholesterol or bis(monoacylglycero)phosphate	Lu et al. (2022)
knockout	immortalized murine macrophage	*in vitro*	NLRP3 inflammasome activation	Schmid-Burgk et al. (2016)
knockout	THP-1	*in vitro*	CD14 expression and macrophage differentiation	Jimenez-Duran et al. (2020)
knockout	ER-Hoxb8	*in vitro*	regulators for proinflammatory alarmins S100A8 and S100A9	Jauch-Speer et al. (2022)
knockout	immortalized human microvascular blood endothelial cell	3D *in vitro*	endothelial cell response to anti-angiogenic bevacizumab	He et al. (2021)
knockout	immortalized mouse aortic endothelial cell	*in vitro*	endothelial cell response to laminar shear stress from the bloodstream	Coon et al. (2022)
knockout	mouse cardiac fibroblast	*in vitro*	reprogramming efficiency of murine cardiac fibroblast into cardiac progenitors	Yu et al. (2019)
knockout	human embryonic stem cell line ES03	*in vitro*	cardiac progenitor formation	Xu et al. (2020)
knockout	mouse cardiomyocytes	*in vivo*	cardiomyocyte maturation	VanDusen et al. (2021)
knockout	human iPSC	*in vitro*	cardiomyocyte replication and polyploidization	Pettinato et al. (2021)
knockout	human iPSC	*in vitro*	doxorubicin-induced cardiotoxicity	Sapp et al. (2021)
knockout	zebrafish embryo	*in vivo*	cardiac development and function	Parvez et al. (2021)

## 4 Discussion

The application of CRISPR technology in cardiovascular research has greatly facilitated the study of molecular basis, disease model establishment and even the development of therapeutic strategies through direct correction of pathological gene elements. CRISPR screening fully releases the power of CRISPR toolkits by allowing comprehensive analysis of massively genetic perturbation at cell, tissue, organ and even the whole animal levels. The studies we discuss here represent pioneering efforts to apply CRISPR screen in cardiovascular research, which significantly accelerate the deciphering of molecular basis underlying cholesterol homeostasis, vascular physiology, inflammation, heart development and regeneration. More importantly, some of the gene hits from these CRISPR screens may serve as actionable targets for developing drugs or therapeutics against corresponding cardiovascular diseases. The CRISPR toolkits are still quickly expanding to offer more powerful and diverse modules for gene manipulation. More efficient delivery methods of these CRISPR apparatus into hard-to-transfect models are highly demanded to broaden the application scope of CRISPR screening in cardiovascular research. The combinatorial use of CRISPR screening and single cell spatial omics in future may help to resolve more complexed questions with advanced *in vivo* models, which is technically unachievable previously but particularly important for cardiovascular research. Given the delicate design of screening readout, appropriate choice of testing tools and models, it is foreseeable that CRISPR screening will play more important roles in cardiovascular research.
